# Meeting of the Members of the Medical Profession of the City of Troy

**Published:** 1854-08

**Authors:** 


*Meeting of the Members of the Medical Profession of the City of Troy.*—A meeting of the members, was held at the Court House, on Friday the 16th inst., to take into consideration the much lamented death of one of their number, Dr. Amatus Robbins.

On motion, Dr. A. J. SKILTON was appointed Chairman, and Dr. TAYLOR LEWIS, Secretary.

On motion, Drs. Blatchford and Brinsmade were appointed a committee, to draft resolutions.

The following resolutions were offered which were unanimously adopted.

*Resolved*, That this meeting learns with the most sincere regret the death of Dr. AMATUS ROBBINS, of this city, who, for the last 40 years, has been in the uninterrupted and faithful discharge of his professional duties.

*Resolved*, That while we sincerely mourn his death, we are thankful to our Heavenly Father that in his merciful goodness, he has so long spared the "beloved Physician,"—to cheer us by his encouragement; to aid us by his advice, and benefit us by his example. And that we seek consolation in humble reliance that his blameless life and the trust which he had in the efficacy of Christ's atonement, has secured for him a blissful immortality.

*Resolved*, That in this dispensation of Divine Providence, we have lost one of our most active, worthy and honorable members, and in the chamber of sickness one of our safest and most reliable counsellors. The loss to some of us seems irreparable.

*Resolved*, That we will attend his funeral, to-morrow afternoon at three o'clock, in a body, and wear the customary badge of mourning.

*Resolved*, That these proceedings be published in our daily papers, signed by the President and Secretary, and a copy sent to each of the Medical Journals in this State, to the Boston Medical and Surgical Journal, and through the Hon. Jonathan Edwards, to the afflicted relatives of the deceased.

At the request of the members, Dr. Blatchford consented to deliver a eulogy on the life of our late and much esteemed brother.

On motion the meeting adjourned.

(Signed.) A. J. SKILTON, *President.*
TAYLOR LEWIS, *Secretary.*

---

SENECA FALLS, June 3, 1854.

DR. HUNT:

*Dear Sir*,—At Dr. Hamilton's request I send you the following facts in relation to my father, Dr. Matthias B. Bellows, who died at Seneca Falls, May 1, 1854.

He was born in Hebron, Washington Co., April 9, 1788: pursued the study of medicine at Little Falls, under the instruction of Dr. Jas. Kennedy, and received a diploma from the Medical Society of Herkimer Co., July 7, 1812. He removed to Seneca Falls immediately after, and began the practice of his profession, which he followed uninterruptedly until disease compelled him to abandon it, being forty-two years of active practice.

In 1844, the honorary degree of Doctor of Medicine was conferred upon him by the Regents of the University, on the recommendation of the State Medical Society.

He was also Curator of the Medical College of Buffalo.

For two years previous to his disease, he had an intermitting pulse, which was attributed to irregular nervous action, and considered a functional derangement merely. He felt so little inconvenience from this, that he continued his practice, and not untill the last year was any organic disease suspected. In November last he was taken with a chill while riding, and expectorated blood freely for forty-eight hours, when it ceased. He became very much excited, and at times delirious. The nervous symptoms being the most prominent, the remedial means were directed more particularly to them. He took Ext. Hyoscyamus and Lettuce, which allayed somewhat the extreme irritability. The constant laboring of the heart was a source of great annoyance to him. He felt the strongest impulse from it, just below the right scapula, but to the examiner it was at the epigastrium. About three months before his death, anasarca commenced in the extremities, which finally became general. From this, after a trial of various remedies, he obtained relief, by the use of the Apocynum Cannabinum, prescribed by Dr. John McCall, of Utica, who made him a visit at this time. He then remained very comfortable for about a month, when it returned worse than before. The stomach became very irritable and rejected the medicine. A breaking up of the left lung occurred to a slight extent, and he raised a large quantity of bloody matter for several days. Ascites was plainly present now, and in a short time serum was poured into the cavity of the thorax.

From the first there was no uniformity between the force of the heart's action and the pulse of the wrist. The latter, weak at first, kept getting weaker, except during the inflammatory action in the lungs, while the pulsations of the heart became more violent and laborious. His common position was in the sitting posture. He suffered much from want of sleep, and when it came, he was constantly dreaming and laboring, and invariably started out of it with a sensation of immediate suffocation. The *bruct de soufflet* which at first was moderate, toward the termination of his disease became very distinct during each systole. There was no pulse perceptible at the wrist for three days before he died, yet the tumultuous beating of heart increased until within a short time of its failing altogether. The contractions of the heart ceased for some moments before respiration.

With respect, I remain,

Yours truly,

JAMES BELLOWS.

---

An active member of our Board of Health found, the other day, a case of cholera in his peregrinations, which seemed to be neglected. He forthwith reported it to his coadjutors.

"Sure its cholera, Col.?" asked the President. "Cholera! of course its cholera. Don't I know cholera? Nose right here!" said the Col., pinching in the alae of his proboscis—"Blue as"—(adjective.)

The cholera case was transported to the hospital. The faithful Sisters put a blister over the stomach, and gave him a tumbler of brandy and water. He took to the latter kindly.

Next day the President inquired of the Col. about his patient.

"Not dead is he?" quoth the Col.

"Reckon not," said the President—"the Sisters stopped his brandy in the morning, and the last they saw of him, he went over a nine foot fence without touching, on his way to the nearest tavern!"

Without intending any reflection on the Col.'s power of diagnosis, we may intimate that his patient was "deeply, darkly, beautifully blue!"

